# Semi-Annual Meeting of the Erie County Medical Society

**Published:** 1863-06

**Authors:** 


### SEMI-ANNUAL MEETING OF THE ERIE COUNTY MEDICAL SOCIETY.

The meeting was called to order by the Secretary, and in the absence of the President and Vice President, Dr. Samo was appointed President *pro tem.*

Minutes of the last meeting were read and approved. The Secretary read a communication from the President, Dr. Winne, to the effect that Judge Davis had not yet rendered decision in the Bartlett case. Himself and Henry W. Rogers had appeared before the Judge and shown reason why Bartlett should not be admitted to the Society as member.

Drs. Winne, Boardman and Gay were appointed Committee to act in concert with a Committee which had been appointed by the City Medical Association for the purpose of obtaining assignment of permanent rooms for the Society in the new buildings proposed to be built for accommodation of the Scientific Societies, Associations, &c., &c., of the City.

*Dr. Gay* presented the subject of holding consultations with physicians who were not members of the Erie County Medical Society, specifying a case of grievance and complaining of it as unprofessional and unfair.

*Dr. Miner* said he had met the physician referred to by Gay in consultation, though he did not presume that he was the physician Dr. Gay was so friendly with, who had met him after another had refused to do so. Was glad the matter had been presented for consideration, and was quite sure that members of the Society residing out of the City were often called and attended patients with physicians who are graduates of respectable colleges, though not members of the County Society. Had himself often held such consultations, both in his office when such physicians brought patients for his advice, and at their own places of residence.—Regretted that there should be any physicians fairly entitled to membership in the Society who should neglect to avail themselves of its advantages.

*Dr. Haunstein* said he had often met the physician referred to by Dr. Gay; that this physician found it so much trouble to get an examination from State censors, he had become vexed and sick of it; especially since this membership afforded little protection in any way, and so few advantages. Said he was unquestionably a capable physician—a very highly educated one. Also remarked that the physician referred to by Dr. Gay as having refused to consult with him on the ground that he was not a member, was himself in company with his father, who was not a member of the Society, and was equally an irregular physician.

*Dr. Gay* was mortified at the confessions of members and regarded it as exceedingly unprofessional for physicians not to become members of the Society, and a still greater disgrace for those who were, to countenance and consult with those who were not, members of the Erie County Medical Society.

Drs. Allen, of Aurora, Barnes, Peters, Whitney and others, participated in the discussion, and a committee was finally appointed to consider the legal bearings of the question and to report at the next meeting what action the Society should take.

Afternoon Session.—*Dr. J. R. Lothrop* gave a very interesting and instructive address upon the "Action and Uses of Alcohol," which was listened to with attention and pleasure by a large number of the members of the Society. This was the only really important feature of the meeting, and was appreciated by every member present.

Delegates to the American Medical Association were called upon for report. Dr. Wyckoff gave an account of the order of exercises, and the various questions which were brought before the Association. The most important reports were those of the Committee appointed to report upon the action of Surgeon-General Hammond in withholding or suppressing the use of calomel and antimony in the army, and of Dr. Cox of Maryland, to improve the standard of medical education.

One well marked and important feature of the meeting was the entertainment provided for members. The evening gatherings afforded opportunity for extending acquaintance with medical men, and was really the most pleasant and attractive part of the meeting, for which the members will feel under lasting obligations to the profession of Chicago.

*Drs. Gay and Nichell* also gave additional account of the Association and of the incidents of the occasion, which interested them most.

*Voted* to adjourn.

